# Step Monitoring to improve ARTERial health (SMARTER) through step count prescription in type 2 diabetes and hypertension: trial design and methods

**DOI:** 10.1186/1475-2840-13-7

**Published:** 2014-01-06

**Authors:** Kaberi Dasgupta, Ellen Rosenberg, Stella S Daskalopoulou

**Affiliations:** 1 Research Institute of the McGill University Health Centre, Montreal, Quebec, Canada; 2 Department of Medicine, McGill University, Montreal, Quebec, Canada; 3 Department of Family Medicine, McGill University, Montreal, Quebec, Canada; 4 Division of Clinical Epidemiology, Department of Medicine, McGill University Health Centre, 687 Pine Avenue West, V-Building (V1.08), Montreal, Quebec H3A 1A1, Canada

## Abstract

**Background:**

With increasing numbers of type 2 diabetes (DM2) and hypertension patients, there is a pressing need for effective, time-efficient and sustainable strategies to help physicians support their patients to achieve higher physical activity levels. SMARTER will determine whether physician-delivered step count prescriptions reduce arterial stiffness over a one-year period, compared with usual care, in sedentary overweight/obese adults with DM2/hypertension.

**Design:**

Randomized, allocation-concealed, assessor-blind, multisite clinical trial. The primary outcome is change in arterial stiffness over one year. The secondary outcomes include changes in physical activity, individual vascular risk factors, medication use, and anthropometric parameters. Assessments are at baseline and one year.

**Methods:**

Participants are sedentary/low active adults with 25 ≤ BMI < 40 kg/m^2^ followed for DM2/hypertension by a collaborating physician. The active arm uses pedometers to track daily step counts and review logs with their physicians at 3 to 4-month intervals. A written step count prescription is provided at each visit, aiming to increase counts by ≥3,000 steps/day over one year, with an individualized rate increase. The control arm visits physicians at the same frequency and receives advice to engage in physical activity 30-60 minutes/day. SMARTER will enroll 364 individuals to detect a 10 ± 5% difference in arterial stiffness change between arms. Arterial stiffness is assessed noninvasively with carotid femoral pulse wave velocity using applanation tonometry.

**Discussion:**

The importance of SMARTER lies not simply in the use of pedometer-based monitoring but also on its integration into a prescription-based intervention delivered by the treating physician. Equally important is the measurement of impact of this approach on a summative indicator of arterial health, arterial stiffness. If effectiveness is demonstrated, this strategy has strong potential for widespread uptake and implementation, given that it is well-aligned with the structure of current clinical practice.

**Trial registration:**

ClinicalTrials.gov (NCT01475201)

## Introduction

Longitudinal studies have demonstrated the protective effects of higher walking levels on arterial health in adults with type 2 diabetes (DM2) and hypertension. In the National Health Interview Survey [1], walking 120 minutes or more each week resulted in a 40% relative mortality rate reduction over 12 years. In the Nurses’ Health Study [2], those in the highest quartile of walking lowered their risk of heart attack, stroke, and related mortality by more than 30% over a decade. However, walking levels have declined in an era of Internet transactions, ‘smart’ phones, and ‘social networking,’ contributing to escalating rates of obesity and its detrimental consequences [3-7].

A meta-analysis indicates that the integration of physical activity promotion into primary care is associated with a 42% increase in self-reported activity [8]. The Green Prescription approach (physical activity-based prescriptions with three telephone-based support calls from trained counselors) has been widely implemented in the primary care context in New Zealand and has demonstrated sustained increases in physical activity levels [9]. These approaches, however, require paramedical staff support which may not be feasible in many settings. A more physician-driven approach had some effect on physical activity levels [10], but physicians generally lack the skills or time to provide more complex physical activity counseling, despite calls for more specific prescriptions [11]. In this regard, step count monitors such as pedometers may be a useful tool.

Pedometers permit simple, real-time tracking of walking and other step-related activity. Among NAVIGATOR trial participants (Nateglinide and Valsartan in Impaired Glucose Tolerance Outcomes Research [12]), both pedometer-assessed daily step counts at baseline (hazard ratio [HR] per 2,000 steps/day 0.90, 95% CI 0.84–0.96) and change in step counts over an average follow-up of 6 years (HR per 2,000 steps/day increase 0.92, 95% CI 0.86–0.99) led to reductions in cardiovascular disease events. These effects were independent of one another and also independent of underlying co-morbidities and changes in body mass index (BMI). Group pedometer-based programs increase physical activity levels and improve vascular risk profiles. In a meta-analysis of eight clinical trials [13], such interventions led to a 2,491 daily step increase [95% Confidence Interval (CI) 1,098 to 3,885], a -3.8 mm Hg change in systolic blood pressure (95% CI -5.9 to -1.7) and a -0.38 kg/m^2^ change in BMI (95% CI -0.05 to -0.72). These programs, however, may not be available or sustainable. Self-monitoring and target setting could be an inexpensive and accessible option, with assistance through web-based tools, but for many individuals this may not provide sufficient accountability and motivational support, elements deemed important to change health behaviours [14].

Integration of pedometer-based monitoring into routine medical visits, however, could help meet the need for accountability and support, without requiring additional staffing. We have developed such a strategy and we are testing it through a randomized controlled trial, as described herein. In our strategy, participants self-monitor and track step counts. They review these records with their physicians who then assist them in setting realistic step count targets, consistent with the monitoring-prescription-monitoring-adjusted prescription dynamic approach to which both physicians and patients are accustomed. Many patients with DM2 and hypertension self-monitor glucose and blood pressure levels; based on this information, their physicians are better-positioned to prescribe and adjust medications [15]. Such a strategy has not previously been evaluated for physical activity monitoring. A recent Green Prescription trial [16] integrated pedometer-based self-monitoring in a clinic setting and demonstrated higher activity levels, but again the intervention structure relied on paramedical clinic staff, rather than being routinely integrated as a component of medical visits with a physician. Thus the physician-patient relationship, an important factor in adherence to medications and physical activity, [17,18] was a less important factor in the intervention. In contrast, this is arguably the most important element of the SMARTER strategy. Another trial [19] did examine a physician-delivered intervention in DM2 and demonstrated a step count increase over a 12-week period but did not examine vascular effects; we are evaluating a one-year physician-delivered intervention and its impact on a summative indicator of arterial health, arterial stiffness, as described below.

The previously-cited meta-analysis of group-based pedometer interventions demonstrated reductions in blood pressure [13]. While blood pressure is one important indicator of arterial health, recent technological developments have allowed for a more comprehensive assessment of the arterial system through measurement of arterial stiffness, capturing the summative impact of vascular risk factors. Epidemiological studies have demonstrated that higher arterial stiffness is associated with increased risk of vascular disease and events [20-24]. In a trial among DM2 patients, a three-month supervised exercise program conferred a 20% greater reduction in arterial stiffness compared to usual care alone [25]. Higher daily step counts have been associated with lower carotid femoral pulse wave velocity (cfPWV), the gold standard measure of arterial stiffness [26,27].

We are conducting a randomized controlled trial to examine the effect of a physician-delivered step count prescriptions on arterial stiffness in sedentary overweight/obese adults with DM2 and/or hypertension. We present herein the design and methods of the SMARTER (Step Monitoring to improve ARTERial health) trial. Our primary objective is to assess the impact of the intervention on cfPWV using applanation tonometry. Our secondary objectives are to assess effects on individual vascular risk factors, medication use, anthropometric parameters, and physical activity, as detailed below.

## Methods

The SMARTER trial is registered at ClinicalTrials.gov (NCT01475201). Funding is through the Canadian Institutes of Health Research (MOPP 114996). Approval of the research protocol has been granted by McGill University’s Faculty of Medicine Institutional Review Board and all participating institutions (McGill University Health Centre, St. Mary’s Hospital, Sir Mortimer General Jewish General Hospital). Written informed consent is provided by all participants.

### Study design

SMARTER is a prospective, randomized, open-label, blinded-endpoint (PROBE) trial comparing two strategies to increase physical activity among overweight/obese adults followed for DM2 and/or hypertension. Allocation is concealed with an intervention to allocation ratio of 1:1.

### Eligibility criteria and recruitment

Inclusion criteria include physician-diagnosis of DM2 or hypertension, age ≥18 years, and 25 kg/m^2^ ≤ BMI < 40 kg/m^2^ to permit accurate pedometer measurement [28]. Exclusion criteria include ≥ 150 minutes of leisure time physical activity per week (i.e., fulfilling physical activity recommendations) [29], co-morbid conditions with potential to affect adherence to trial procedures (e.g. inflammatory arthritis, active malignancy, major depression or other significant psychiatric disorders, and/or significant visual impairment), and pregnancy/planning a pregnancy. Further, following baseline evaluation but prior to randomization, those with a baseline step count averaging >10,000 steps/day (i.e., active) [30] are excluded. Treating physicians are free to modify medications during the trial period in both arms. Collaborating physicians are based at family medicine, internal medicine, endocrinology, and hypertension clinics in Montréal, Canada. They identify potentially eligible participants during routine clinic visits and obtain assent for contact by SMARTER personnel.

### Measurements

All measurements detailed below are assessed both at baseline and following the intervention at one year so that changes during the trial may be computed. These evaluations are conducted at our Vascular Health Unit (Division of Internal Medicine, McGill University Health Centre).

#### Arterial stiffness

To avoid circadian variations that may affect arterial stiffness measurements, we evaluate all participants in the morning at approximately the same time of the day at baseline and again at the final evaluation. Participants are instructed to abstain from: i) caffeine and ethanol intake for at least 12 hours and flavonoid-containing foods (such as berries, grapes, apples, green tea, chocolate, nuts, herbs, and spices) for at least 24 hours before the evaluation, ii) any strenuous exercise (aerobic or anaerobic) for 24 hours before the evaluation, and iii) exposure to cigarette smoke for at least 12 hours before the evaluation. The participants are instructed to take all their medications except their antihyperglycemic medications in the morning of the baseline and final evaluations. Participants are requested to fast overnight (12 hours) prior to evaluation.

CfPWV is measured using applanation tonometry through the SphygmoCor system [31-34]. A high-fidelity micromanometer is placed on the tip of a hand-held tonometer (SPC-301; Millar Instruments, Houston, TX, USA) and applied to the skin overlying the radial artery to flatten but not occlude the artery. Using a previously validated generalized transfer function, the system software calculates an averaged radial artery waveform and derives a corresponding central pressure and other indices of pulse wave analysis, including the augmentation index and augmentation pressure [31,32,35,36]. The tonometer is then applied over the carotid and subsequently the femoral arteries with concurrent 3-lead ECG monitoring; the PWV is automatically calculated from measurements of the pulse transit time and the distance between the carotid and femoral recording sites [PWV = distance (m)/transit time (s)].

#### Physical activity

Participants are provided with two pedometers (Yamax SW-701; viewing windows concealed) to assess step counts and an accelerometer (Actiwatch-2; Phillips, Respironics) to capture duration, frequency, and intensity of physical activity. Participants wear one pedometer and the accelerometer for one week and then mail these to the study centre with the unused pedometer (padded, pre-addressed, pre-stamped envelope). The unused pedometer captures the “postman steps” that occur during the mailing process [37-39]; these are subtracted from the step counts recorded on the pedometer that is worn. Steps/day are computed from this corrected value.

#### Physical fitness

Cardiorespiratory fitness is assessed through a supervised maximal incremental test to determine VO_2max_ (model VMax229LV, Sensorsmedics, Yorba Linda, CA, USA) with treadmill testing (MedTrackCR60 Treadmill, Quinton, Bothell, WA, USA; Bruce ramp protocol).

#### Anthropometric measures

Weight and height are assessed to the nearest 0.1 kg (SECA 882 electronic scale, light clothing, shoes removed) and 0.1 cm (stadiometer), respectively. BMI is calculated by dividing the weight in kilograms by height in metres squared. Waist circumference is measured midway between the iliac crest and the lower rib margin. Hip circumference is measured at the point of greatest posterior extension of the buttocks.

#### Blood pressure

Resting blood pressure is measured using the BpTRU Blood Pressure Monitor using a standardized protocol [40].

#### Serum biomarkers

##### 

**Lipid profile** Total cholesterol, high density lipoprotein cholesterol (HDL-C), and triglyceride levels are measured using spectrophotometry and low density lipoprotein cholesterol (LDL-C) is calculated (Friedewald equation). Apolipoproteins A1 and B are measured using the turbimetric method. In participants with DM2, *hemoglobin A1C* is measured with a high-performance liquid chromatography (HPLC) analyzer [41]. In those without DM2, fasting glucose and insulin levels are measured and the Homeostatic Model Assessment is calculated [(fasting insulin in μunits/ml X fasting glucose in mmol/L) ÷ 22.5] [42-47]. *High sensitivity C-reactive protein* is assayed through an immunonephelometric method.

#### Cardioprotective medications

Use of antihypertensive, antihyperglycemic, and lipid-lowering medications (number and dose) is recorded.

#### Adherence

We are tracking clinic visits completed, prescriptions written by collaborating physicians, and use of the step count log book.

### Randomization

Patient inclusion and data entry forms (eCFRs) were created using web Electronic Data Capture software platform from Dacima™. Data are entered through a web browser into a web database that complies with regulatory requirements (FDA 21 CFR Part 11). First, data from the initial telephone-based eligibility screen are entered on the electronic eligibility check web form. The EDC system verifies the candidates’ responses to make a preliminary determination of eligibility. Candidates who fulfill preliminary assessments for eligibility present in person for informed consent procedures and baseline assessment. The baseline evaluation is considered completed once the pedometers with concealed viewing windows are received by study personnel (see **Measurements**, *Physical activity*). Pedometer-based step count data are entered into the web form. Those with more than 10,000 steps/day are determined to be ineligible by the EDC inclusion algorithm and marked as excluded.

Eligible individuals are randomized to either the control or the active trial arm through the Dacima™ Clinical software (i.e., individual-level randomization with no stratification; random permuted blocks with randomly-varied block sizes of two, four and six).

### Interventions

Participants are followed by their physician at 3 to 4-month intervals over 12 months. The control arm receives advice to engage in 30-60 minutes of activity daily, consistent with usual care [48]. In the active arm, the physician writes a step count prescription at each visit. As previously noted, pedometer-based interventions led to a 2,491 daily step increase in a meta-analysis [13]. A step count increment of 2,500 to 3,000 steps is roughly equivalent to 30 minutes of walking at a moderate pace, as established through direct counts of individuals walking on a treadmill at a workload of 3 metabolic equivalents (METS)/minute [49]. In the SMARTER active arm, the aim is to achieve a net increase over baseline values of at least 3,000 steps/day over one year. DM2 and hypertension follow-up visits are recommended at 3 to 4 month intervals. Testing an intervention over 1 year permits 3 to 4 contacts with the treating physician but still respects the reality of usual clinical follow-up. This is important if the results of this trial are to be implemented in real-world practice following the trial itself. A 1-year study duration will mean that participants are examined at roughly the same time of year (i.e., season) at the baseline and final assessments. This will help ensure that any changes demonstrated are not attributable to seasonal differences [50]. Finally, many physical activity intervention studies have been criticized as being too short in duration; we suggest that a 1-year period of intervention and follow-up provides a reasonable time frame over which to evaluate effects.

**Figure 1:**
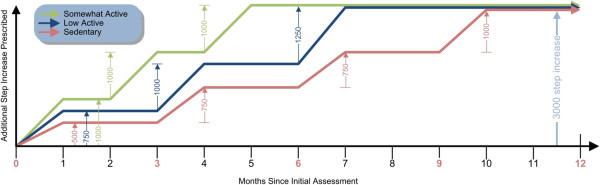
**Step count prescription framework by baseline activity category.** Months in red are those at which a clinic visit occurs. All participants should ultimately achieve a net increase of at least 3,000 steps/day from baseline, as indicated in light blue.

The rapidity at which this is achieved is individually-tailored (Figure 1). The baseline step count value is provided by the research team as derived from the baseline assessment. The general time frame for the increase by 3,000 steps/day is 10 months for sedentary participants (<5,000 steps/day), 7 months for low active participants (5,000-7,499 steps/day), and 5 months for somewhat active participants (7,500-9,999 steps/day). Subsequent prescriptions are based on step counts achieved and individual circumstances.

### Sample size

Madden and colleagues [25] conducted a randomized controlled trial comparing a 3-month supervised exercise program with usual care in adults with DM2, hypertension, and dyslipidemia. Exercise sessions were three times per week (cycle ergometers, treadmills). They demonstrated a 13.9% reduction in cfPWV in the intervention arm compared with a 4.4% increase in the control arm (i.e., an 18.3% difference). To be conservative, we aim to detect a 10% difference between our active and control trial arms to an accuracy of ± 5% over a one-year period. This implies that our CI for the difference will be narrow enough to rule out only trivial effects. Based on the report by Madden and colleagues, we estimate a standard deviation (SD) of 28 in the active treatment arm [standard error, SE* sqrt (n) = 6.7 * sqrt(17)] and 14 for the control arm [3.3*sqrt (17)]. Under these conditions, with ± 5% CI width, we will need to retain 151 per arm (i.e., 302 in total). Allowing for a loss to follow-up of up to 17% based on our previous studies, [50,51] we require a sample size of 364 individuals (i.e., 182 per arm).

### Statistical analysis

Our main outcome is percentage change in arterial stiffness (i.e., cfPWV) within each trial arm. We will calculate mean between trial arm differences in “after minus before changes”, with 95% CIs (intent-to-treat analysis). In addition to examining between trial arm differences in percentage change from baseline arterial stiffness, we will also evaluate between arm absolute differences in raw “after minus before changes” in cfPWV. Although trial arms are likely to be similar at baseline, we may use a linear model to adjust for any possible differences between arms for important covariates (e.g., baseline daily steps, baseline BMI, age, sex, changes in medications). If relevant, in a sensitivity analysis, we will use multiple imputation to impute missing data. A similar approach will be used to examine between-arm differences in daily steps and vascular risk factors. We will also correlate increments in steps to changes in arterial stiffness, anthropometric parameters, and specific vascular risk factors. Finally, we may employ additional secondary analyses (e.g., per protocol analyses; analyses in DM2 alone, hypertension alone, DM2 and hypertension; analyses in participants with and without medication changes).

## Discussion

The SMARTER trial is testing an innovative step count prescription strategy delivered by physicians to their patients with DM2 or hypertension. The focus on daily step counts arguably simplifies the process of physical activity prescription. Increasingly, physicians and patients are partners in monitoring and managing chronic diseases. Such partnerships can improve patient adherence and health outcomes [15]. Less than one third of North Americans achieve the recommended 10,000 steps/day [52] and we have demonstrated that in adults with DM2 and/or hypertension, mean counts are low at approximately 5,000 steps/day, with a further 15% reduction during fall and winter months [50]. “Re-engineering” walking into daily life could be an effective means of improving the arterial health of patients with DM2 and/or hypertension.

The effects of the SMARTER intervention are being determined in terms of biological impact, particularly in terms of changes in arterial stiffness as captured by cfPWV. In the Framingham Heart Study, a one SD increment in arterial stiffness was associated with a 48% increase in arterial disease risk, independently of individual vascular risk factors [23]. A meta-analysis indicates that an increase in cfPWV by 1 m/s corresponds to an age-, sex-, and risk factor-adjusted risk increases of 14%, 15%, and 15% in total cardiovascular disease event rates, cardiovascular mortality, and all cause mortality, respectively [53]. A recent analysis of the Framingham Offspring study demonstrated that higher aortic stiffness is associated with higher risk of incident hypertension [24], suggesting that in some cases stiffness precedes the development of hypertension. Illustrating the responsiveness of cfPWV to higher activity levels, a trial among older patients with vascular risk factors including DM2 demonstrated a 3-month supervised exercise program to confer an important reduction in cfPWV compared to usual care alone [25].

We are aware of some potential limitations to the trial. Firstly, physicians cannot be blinded to the intervention given that they are delivering it. However, outcome assessors are blinded to intervention arm status, and both the primary outcome and most of the secondary outcomes are evaluated objectively (i.e., assays or automated measurements). We acknowledge the possibility of contamination of the control arm: that is, physicians may be tempted to employ the SMARTER intervention in the control arm, particularly if they perceive benefit. However, only the active trial arm receives pedometers at the onset of the intervention period. Following the final trial evaluation, the control trial arm participants will be provided with a pedometer in gratitude for their participation, and they will be free to engage in a step count prescription strategy with their treating doctor. This ‘delayed’ intervention may facilitate adherence to study procedures. Physicians are free to modify medications during the trial period and this could impact some outcomes. To address this possibility, we will perform secondary analyses with (i) adjustment for changes in medications and (ii) restriction to participants without such changes. We acknowledge that more frequent contact with physicians and/or other health care team members could strengthen the impact of the intervention; however, DM2 and hypertension follow-up frequently occurs in settings without multidisciplinary teams and the demands of clinical care may render more frequent contact challenging. We have, therefore, designed our intervention to be ‘pragmatic’ with 3 to 4 follow-up visits over one year accompanied by a written prescription. Patients with DM2 and hypertension generally have common features including insulin resistance and increased risk for vascular disease. The inclusion of participants with DM2 and hypertension, each alone or in combination, allows us to achieve recruitment targets in a reasonable time frame and to generalize our results to groups of patients seen frequently in clinical practice; we will nonetheless perform subgroup analyses (DM2 alone, hypertension alone, DM2 and hypertension in combination).

We have developed our trial in collaboration with physicians who manage DM2 and hypertension. We held several meetings with over 24 physicians to discuss the intervention. They underscored their need for physical activity promotion tools and viewed the SMARTER strategy to be feasible and easily integrated into clinical practice. They, nonetheless, expressed the need for Level A evidence as derived through a randomized controlled trial. This integrated knowledge translation approach will facilitate future knowledge translation efforts. If effectiveness is demonstrated, we are well-positioned to consider inclusion in Clinical Practice Recommendations for DM2 and hypertension.

The SMARTER intervention is novel in several respects: (i) It is physician-specific intervention that may be particularly important in understaffed clinics without the support of a full multidisciplinary team. (ii) A specific, signed, stamped written prescription is provided, focusing on step count targets. (iii) The rate of step count titration is individualized, based on baseline step counts, leveraging an existing therapeutic alliance. (iv) The primary outcome is change in arterial stiffness, a summative indicator of arterial health. Prior pedometer-based intervention studies have examined specific individual vascular risk factors but to our knowledge, none have evaluated a summative indicator of arterial health (arterial stiffness) as the primary outcome. (v) A shortcoming of prior studies has often been a lack of attention to medication use. This is being carefully recorded during our trial and we will perform subgroup analyses in those with and without medication changes (e.g., antihypertensive agents, antihyperglycemic agents, lipid-lowering agents). We will further evaluate changes in medication use as a secondary outcome. (vi) This will be one of the largest pedometer intervention trials ever conducted (364 participants).

In summary, the potential impact of the SMARTER intervention relates not only to the use of pedometers but rather to its incorporation into clinical care, leveraging the physician-patient therapeutic alliance. A key strength of the SMARTER trial is the ability to capture biological impact through measurement of arterial stiffness. Demonstration of effectiveness of the SMARTER intervention has strong potential to lead to wide and sustained adoption in clinical practice with integration in DM2 and hypertension management.

## Abbreviations

BMI: Body mass index; DM2: Type 2 diabetes; CI: Confidence interval; cfPWV: Carotid femoral pulse wave velocity; PWA: Pulse wave analysis; PWV: Pulse wave velocity; ECG: Electrocardiogram; VO2max: Maximum oxygen uptake; HDL-C: High density lipoprotein cholesterol; LDL-C: Low density lipoprotein cholesterol; Hemoglobin A1C: Glycosylated hemoglobin; HPLC: High performance liquid chromatography; MET: Metabolic equivalent; CVD: Cardiovascular disease

## Competing interests

The authors declare that they have no competing interests.

## Authors’ contributions

KD and SSD conceived and designed the study and co-wrote the study protocol, with critical input from ER and the SMARTER collaborators. All authors read and approved the final manuscript.

## Author’s information

KD and SSD are Physician Scientists and Associate Professors in the Department of Medicine at McGill University. ER is Associate Professor in the Department of Family Medicine at McGill University. KD and SSD both hold clinical investigator salary awards from the Fonds de recherche Santé du Québec. KD additionally holds the Société québécoise d’hypertension artérielle-Jacques de Champlain Award; SSD is a previous recipient of this award.
